# The Implementation of a Remote Work Program in an Italian Municipality before COVID-19: Suggestions to HR Officers for the Post-COVID-19 Era

**DOI:** 10.3390/ejihpe11030064

**Published:** 2021-08-15

**Authors:** Salvatore Zappalà, Ferdinando Toscano, Gabriela Topa

**Affiliations:** 1 Department of Psychology “Renzo Canestrari”, Alma Mater Studiorum University of Bologna, 40126 Bologna, Italy; ferdinando.toscano@unibo.it; 2 Department of Psychology and Human Capital Development, Financial University under the Government of the Russian Federation, 125993 Moscow, Russia; 3 Department of Social and Organizational Psychology, National University of Distance Education, 28040 Madrid, Spain; gtopa@psi.uned.es

**Keywords:** remote work, telework, public sector, human resource management, change management

## Abstract

This case study describes the implementation stages and some outcomes of a remote work program that was adopted in an Italian municipality before the COVID-19 pandemic. This research used a qualitative case study approach, proposing a semi-structured interview with 14 staff members (six remote-worker employees, their respective managers, and two intermediate-level managers) about the experience with the remote work program. In addition, two researchers attended two preparatory program meetings. The evidence shows that, even before the COVID-19 pandemic, remote work was mainly performed at home, for one or two days a week. Together with their manager, remote workers decided the tasks to perform remotely and the criteria to monitor remote work. Furthermore, employees appreciated the remote work program, perceiving themselves to be more productive in their work. Elements of this case study may be relevant for companies that aim to move from an emergency to a more planned remote work.

## 1. Introduction

Remote work has often been conceptualized and adopted in many companies, as a way to improve employees’ performance and wellbeing, and reduce work–family conflict and environmental pollution [1,2,3,4].

In Italy, the flexible work arrangements that characterize remote work have been called “agile work” (“*Lavoro agile*”) or “smart working”, and are regulated by law number 81/2017. This law gives public and private sector employees the possibility to work (1) in other places, beyond their office, and (2) at other times of the day, beyond the official working hours.

Data report that before the COVID-19 pandemic the adoption of remote work in the public administration across Europe was slower than in some areas of the private sector [5]. Similarly, in Italy, the introduction of remote work in the public sector was modest. In 2019, just before the pandemic, only 16% of Italian public bodies were offering agile work opportunities to some of their officers [6].

The health emergency due to COVID-19 forced organizations to ask many employees to work from home, to reduce the spread of the infection. This emergency accelerated the adoption of remote work and required skipping many of the steps that are necessary to implement an efficient remote work program [7]. The result was a so-called “emergency telecommuting”, quite different from the planned choice made by organizations when launching remote work programs. However, setting up a flexible and productive remote work program is not an overnight journey. For instance, an efficient smart working program requires that managers develop new procedures to monitor and supervise employees, while remote workers learn how to coordinate with managers and colleagues [8]. Furthermore, context-specific barriers must be overcome, especially in Italian public administration. In these organizations, the lack of awareness of remote work benefits (partially recognized during the pandemic crisis) and the limited digitization of tasks [6], make it more challenging to accept and adopt remote work, and require interventions that need time and effort.

Although COVID-19 urged organizations to skip the preparatory aspects of remote work programs, planning the introduction of a new program is essential. The literature suggests focusing on the procedures adopted by organizations to achieve efficient remote work programs [9].

Therefore, this case study describes the organizational choices and procedures that managers and employees of an Italian public administration adopted during the remote work program pilot test that was launched before the COVID-19 crisis. The purpose of this case study is to describe a successful experience, so that managers and human resource (HR) professionals can gain insights into how to implement a remote work program in a planned, non-emergency way.

After a short introduction on remote work, describing the challenges for public administrations and the results observed in the literature, the paper illustrates the procedures that the municipality adopted to set up and monitor the performance of remote workers. We describe what participants reported about the project’s main results. In the Discussion section, we elaborate on the change that has been promoted by the remote work program and the implications for public HR managers grappling with a redesign of remote work procedures in the post-COVID-19 era.

## 2. Remote Work: Main Outcomes and Challenges for Public Administration

The literature on flexible work arrangements describes many different ways to name “agile work”, as, for instance, telework, telecommuting, flexible work, remote work, or mobile work [9]. The Italian legislation defines agile work as the possibility to use information and communication technology to work in many different places (e.g., another branch of the company, employees’ home, clients’ premises, teleworking hub, or public spaces, such as public libraries) and/or to work in different moments of the day and the week than those usually used at their office [10].

Recent reviews, such as those conducted by Allen, Golden, and Shockley [9], or Charalampous and colleagues [11], suggest that, in general, telework is related to increased job satisfaction, organizational commitment, and work performance. It shows promising results both for organizations that, for example, benefit from reduced turnover intentions [12] and the employees, who show enhanced psychological health, which, in turn, reduces sick leave [13].

Remote work, however, appears to be not only associated with benefits, but also with several challenges for the managers and staff of public institutions. A first challenge is that traditionally employees are managed and controlled when they are in the workplace, where they interact and coordinate with their manager and colleagues [14]. Remote work diminishes the possibility of supervising employees and requires new practices to facilitate their control [8].

A second major challenge concerns the management, from one side, of non-teleworkers employees and, from the other side, of employees allowed to work remotely. The former may experience dissatisfaction because they may wish to profit of the remote work opportunity, but their work does not fit remote work, or their manager does not allow them teleworking [15]. In general, several roles and positions cannot be covered remotely. On the other hand, many employees are interested in this type of arrangement and, in some cases, they constitute special groups of employees as, for instance, young women [16], millennials [2,17], or older workers [18]. In addition, because remote work requires information and communication technologies, managers should also consider how much the organization is ready to adopt such technologies and how much remote workers are familiar with such tools. In fact, the effectiveness of technology is related to the personal skills and characteristics of the users, and the context in which it is used [19]. This issue, valid in any context, is even more relevant in the Italian public sector, in which many employees are described as not well accustomed to the use of digital technology [20]. In other words, public institutions should consider all these different groups, and develop inclusive processes that are capable of potentially enabling everyone to work remotely and efficiently.

A third challenge that managers of public administrations should consider is that agile work creates professional isolation from colleagues, the organization, or office events. This social isolation has been extensively observed in teleworkers of the public sector [21,22] and has further intensified during the COVID-19 pandemic [23].

Since flexible work arrangements involve individual, work, and organizational aspects [10], a broad point of view should be adopted in analyzing its implementation. Considering that Taskin [24] suggested to conduct qualitative studies for examining the contextual factors impacting the introduction of an agile work program, this exploratory and descriptive study investigates the procedures that a municipality followed when launching a remote work program. The study aims to answer the following three questions:

(1) Which are the main locations, when, and how often, agile workers perform their work duties in a non-emergency period?

(2) How do managers and agile employees select and monitor tasks to be performed remotely?

(3) Which are the personal and collective outcomes of a non-emergency remote work program reported by agile workers and their managers?

## 3. Methods

### 3.1. Participants and Procedure

The case study reported here was conducted in a municipality that decided, well before the sanitary emergency, to implement a remote work project. The municipality is located in Northern Italy. The first two authors conducted a semi-structured interview with 21 employees; six of them (4 F, 2 M; average age: 48.3; average tenure: 14 years) had been selected as remote workers for a pilot program of six months. The other 15 participants in the study were indirectly involved in the smart working project because they were managers or colleagues of the smart workers. Among them, eight were managers or supervisors of the remote workers (six were the sector managers, two were the service managers—an intermediate-level manager between the employees and the sector manager), and seven were colleagues of the agile workers. The first two authors also attended two preparatory meetings in which HR officers and the employees participating in the pilot program discussed the organizational and technical aspects of the project.

An ad hoc committee had selected the employees participating in the remote work project out of a more significant number of candidates. Each candidate had to match one or more of the following criteria: (1) need to take care of their health (for chronic or disabling illnesses), (2) need to take care of a family member (children or elderly parents), and (3) commuter status (long distance from work). The six selected remote workers worked in different departments and, except two of them, had different tasks, roles, and professional backgrounds. The primary function and duty for each person are as follows: (1) engineer, examination of building construction practices; (2) engineer, inspection and design of buildings; (3) accountant, financial accounting; (4) social worker, social assistance; (5) librarian, cataloging historical photos; (6) surveyor, road maintenance. Three smart workers were interviewed at the beginning of the agile work experience, and the other three workers after three months since the start of the program, to have a comprehensive perspective of the different steps of the remote work program.

The management of the municipality and the Department of Psychology of the University of Bologna approved this project. The interviews were carried out in agreement with the Helsinki Declaration (and subsequent revisions) and the Italian regulations on data protection and privacy. All participants gave their informed consent before starting the interview.

### 3.2. Research Design and Instruments

A descriptive qualitative case study design was used for this study [25], which describes the implementation of an organizational change intervention and focuses on “how” a remote work program unfolded and which outcomes it pursued. Such research design is recommended when examining a phenomenon in its specific context [26].

Although the study concerns only one organizational context, it describes workers fulfilling different tasks, working in different offices, and having different managers and colleagues; thus, each smart worker can be considered a replicated case under different conditions [25].

The semi-structured interviews were slightly different depending on the interviewee’s role (smart worker/colleague/manager). The interviews with the smart workers covered the following topics: (1) tasks and activities carried out when working remotely; (2) reasons to participate in the program and consequences in the office because of the request (in terms of re-organization or re-distribution of tasks, impact on colleagues, or on customers); (3) (personal and organizational) advantages and disadvantages expected from the agile work arrangement and actually observed.

In this study, we considered only smart workers’ and managers’ perspectives because they were primarily involved in the design and monitoring of tasks to be carried out in remote work and in assessing personal and organizational outcomes. Interviews with colleagues of remote workers were mainly focused on the interpersonal consequences that the smart working program was having on the relationships within the offices, which is an aspect less pertinent with the aims of this study.

### 3.3. Data Analysis and Research Questions

The researchers verbatim transcribed the interviews with the six agile workers. The answers were screened and summarized in tables to identify statements and evidence related to the study’s purposes. The factual events mentioned during the interviews (e.g., where and when remote work was conducted, how it was monitored, reasons to participate at the program, outcomes of the program, and other actions and events reported by the six smart workers) were examined in relation to each research question and then summarized and reported in three different paragraphs, one for each purpose of the paper. Keeping in mind the information provided by the remote workers, we examined the statements of the managers, checking if managers’ statements confirmed, clarified, or provided different information from those provided by employees. The use of two informants (remote workers and their managers) aimed to increase the internal validity of the evidence reported here [25].

We report some literal sentences to describe the experience of the smart workers. After each literal sentence, we indicate a fictitious name and the respondents’ role, gender, and age.

The following three sections report findings related to each research question. They examine the following: (a) when and where employees worked remotely, (b) how tasks to be conducted remotely were selected and monitored, and (c) the perceived outcomes of the remote work experience.

## 4. Smart Working: How Often, Where, When

In the pre-COVID-19 scenario in which these employees experimented with remote working, different arrangements were possible along the two “flexibility lines” of when and where to work. Analyzing “when” the work outside the organization took place, five employees of the municipality reported to work two full days a week in smart working, while one employee organized smart working in a “transversal” way—she worked remotely every day, for a small part of the working day.

*“Usually, I start to work at the office at 8:00 a.m. and finish work at 2.00 p.m. but, when I am in smart working, I leave the office at 1:00 p.m., which means I have one hour of work left. I do that hour of work in the afternoon. Thus, I organize myself, and at home I establish when I will work that remaining one hour, trying to avoid doing less or more work. However, it is easier to work more, than less, of one hour”.*

(Vittoria, Social worker, F, 41)

Beyond choosing whether to work remotely on a whole working day or only part of it, the interviewed employees tended to organize their daily working time into blocks to be carried out during the day. Indeed, remote workers distributed their tasks throughout the whole day, rather than working 6.50 h consecutively, as typically done in the office. Some examples are as follows:

*“Immediately in the morning, at around 7:00 a.m., I start to work… for two or three hours… I work this way because luckily everyone at my house gets up very late [the worker lives with her old parents] so I take this opportunity to do some work in this period. Then, the whole chaos of breakfast, preparing lunch, shopping at the supermarket, and so on, begins. After lunch, I do some more work, and then it depends. Sometimes, I work all afternoon, sometimes I work three hours and then I take out my 92-years old father for a walk, completing my hours after dinner”.*

(Michela–Librarian, F, 63)

*“I usually work for one hour, early in the morning, before accompanying my children to school. Then, once back home, I work until one o’clock. Then, if I need to finish what I have to do … I work in the early afternoon or when I can”.*

(Lorenzo–Engineer, M, 43)

The municipality did not force all agile workers to work the same amount of hours per week when in “smart” mode. Two of the interviewed employees reported working remotely 7–8 h per week (about one day a week), while the others reported working 12–15 h per week (about two days a week). These employees and their managers reported that they decided how many days working remotely after excluding periods when the office was open to the public (if required by the service) and by taking into account both the personal needs of the colleagues or specific office situations (e.g., people leaving the staff due to retirement).

When considering the locations, that is, “where” to work, all the interviewed persons mentioned their house as the preferred place, although other places were used in specific situations. The following is an example:

*“I usually work from home. However, in this period, I am using the public library in the town where I am for three weeks, and where my family [wife and two children] are having holidays. Needing an internet connection, I work in that library, although it is not very convenient for work … because I have to use my mobile phone, and because it is necessary to respect silence, I have often to go out and talk outside the library room. However, it is a temporary, summer, situation”.*

(Marco–Engineer, M, 43)

Some agile workers expressed no preferences for working in a specific space of their own home, while others reported having favorite places of work, which also changed during the day, such as the dining room, their bedroom, or the terrace. The flexible arrangements here seem to confirm that smart working increases work liquidity, enhancing work performance’s temporal and spatial discontinuity.

## 5. Smart Working: Tasks Selection and Monitoring

The remote workers of the municipality unanimously stated that they wrote down a proposal of the tasks to be carried out during the smart working; this proposal was discussed with the manager, and, together, they established the final list of the tasks to be performed. The managers confirmed this process during their interviews. In some cases, the decision was simple, and managers only had to ratify what the remote worker proposed. In other cases, managers reported that the process was not easy, and required an analysis of the activities and procedures of the whole office. The interviewed employees also noted that the human resources office of the municipality, in charge of implementing the smart working program, was also involved in this process, sharing information on the job design and work processes of the remote workers and the office in which they worked.

Simply put, employees without contacts with the public or external stakeholders, essentially carried out remotely the same tasks that were usually carried out in the office. Conversely, employees interacting with the public or dealing with office activities not requiring a computer, had to consider a narrower range of activities to be performed remotely.

To carry out their tasks remotely, employees reported spending more time planning their work activities. The tasks to be carried out outside the office were planned the day before, when they were in the office, and vice versa. This advanced planning stimulated, in some circumstances, a more general reflection on work activities, adopted methodology, and potential enrichment of the offered services.

*“I now have to work from here [the office] also with a view to what I will have to do at home. And so I always have to manage my files a little more carefully […] as I tell you, many activities are not strictly divisible between those done in smart and non-smart working […] and so many times I have to prepare some things I will do at home and vice versa … because there are activities that develop in several days and have to be continued even when I work away from the office”.*

(Loredana–Accountant, F, 54)

A crucial aspect of smart working—and a concern for many managers and opponents of this arrangement—is the monitoring and evaluation of the job performance of remote workers. The variety of activities, roles, and tasks that are performed by the employees that we interviewed resulted in the design of many forms to report daily activities. These forms consisted of (1) a daily report that was fillable in a spreadsheet, available in a cloud platform and accessible to the manager, whose basic structure was similar for each employee involved in the pilot program; and (2) a detailed report, monthly filled, specific and personalized for each role and position. Employees had to fill both of them.

The generation of forms and performance indicators, and the identification of the activities to perform remotely, resulted from the collaboration between employees and managers that worked together, revising a list of indicators drafted by the employees. The employees reported that, also in this case, the HR office contributed and supported the definition of the indicators, suggesting possible outputs of activities, such as the number of practices instructed or the number of written reports.

Employees complained that the quantitative nature of the monitoring forms made it more challenging to report details of the work done, especially for less routine or specific activities. For example, the librarian in charge of cataloging the photos said that the numerical indicator of the cataloged images was hardly “indicative”, given the wide range of details that can be entered for each shot. For example, a photo can be cataloged by only describing the picture (e.g., “A famous painter with two other people”) or trying to understand, through further research, the identity of the other two people portrayed. Thus, from one side, reporting simple numerical indicators gave rise to doubts in the agile workers. They wanted to further specify the effort made during the day, for instance, adding the procedures carried out to complete the job. On the other side, the selection and refinement of indicators was an ongoing process. Some employees reported that completing the monitoring forms originated reflections that resulted in the revision of the document, making it, in the end, more appropriate to the specific tasks to be performed.

*“Initially, we thought of indicators concerning the average time necessary to process the documents related to the buildings under examination. Lately, we have been looking at things a little better, and I proposed to report not only average time, but the maximum time of elaboration of the proceedings because it is the essential element. The manager confirmed that this proposal was okay, and my colleagues agreed, too… Therefore we are now going ahead in this direction”.*

(Lorenzo–Engineer, M, 43)

The reflection of employees on their duties and monitoring of these duties led to personal initiatives, which, although limited, can be considered as job crafting actions. Such initiatives constitute an attempt to adapt procedures or work relationships to make them more functional [27]; such initiatives are also generally associated with higher job performance [28].

During the interviews, managers stated that they were satisfied with the monitoring system that was developed. They found the indicators and forms to be effective instruments to monitor employees. Managers even positively evaluated the revision of the indicators carried out by employees.

## 6. Experienced Outcomes

The reports that were used to monitor the employees’ performances confirmed managers’ impressions that the output of remote work activities was, in half of the cases, comparable in quantity and quality to that one observed in the office. In the remaining cases, the managers reported that the work performed remotely was better than that one performed in the office, in terms of the quality, quantity, and execution speed. The employees also noted that, when working remotely, they felt that they were working better, faster, and more reactively. The perception of having fewer interruptions was unanimous. However, disruptions of a more “domestic” nature, such as requests from family members, sometimes replaced office interruptions, such as calls or requests by citizens and/or colleagues.

Moreover, almost all smart workers expressed their feeling of being “empowered” by agile work. This was attributed to: (a) the perception of trust by superiors who offered them this arrangement, and (b) the increased attention that they dedicated to the activities carried out remotely, the protection of citizens’ privacy, and the equipment that they received. To protect the intranet system, all remote workers involved in the project received, by the municipality, a laptop where all the specific software used at the office, and updated antivirus, were installed.

*“I’m happy with my relationship with the organization. This fact gave me a certain … I don’t know … satisfaction for being selected for this smart working program, which I consider as a demonstration of trust towards me. In the sense that, if I were a person who does not deserve trust, they would never select me for the smart working, would they? That is, over the years, there have been cases of people that would probably be not selected for the smart working. From a certain point of view, the fact that I was chosen is making the relationship with the organization quite gratifying”.*

(Loredana–Accountant, F, 54)

In addition to the results related to work, employees mentioned other outcomes, such as having more time for family and home-care, lower perception of wasted time (e.g., due to commuting), and psychological (a sense of calm, serenity) and improved physical health (more “energy”, more breaks during work, more responsiveness, and less exposure to cold weather when commuting). Instead, the remote workers rarely encountered the disadvantages that they feared before the start of the pilot program. The expected, but not occurred, disadvantages concerned a decrease in the quantity and quality of relationships with colleagues, an “invasion of work at home”, or “never stop working”. Nevertheless, some employees reported having continued to think about their tasks even when they were not working.

*“Surely, you work better because you have no type of interference, so no phone ringing, no colleague asking … The problem is that I get lost in my work, so I do not look away from the monitor for endless hours. […] When there are deadlines, if I were in the office, when my time is finished, I quit and go away, but instead at home I continue to work even after the official time … so I have to control myself not to work overtime”.*

(Maria–Surveyor, F, 46)

No significant changes in the relationship with colleagues were mentioned, except for aspects that were defined as minor, recoverable, such as rumors unrelated to work that the smart workers heard a few days later. The limited effect that smart working had on interpersonal relationships with colleagues probably depended on the little time spent working remotely (only one or two days a week) and the fact that smart workers were, anyway, frequently in contact with colleagues through telephone and e-mail. Employees and managers also reported that the relationships between smart workers and supervisors were as good as ever.

Finally, two employees reported that colleagues of other departments, who seemed to question the quality and quantity of the work performed during smart working, mocked them.

*“I do not know how much the others who do not do agile work can “trust”, so to speak, this way of working… In the beginning, I had people who made jokes; in short, they let us understand that I could be one of those persons that, once at home, do whatever they want… Smart working is based a lot on trust and if we have to think that employees accept that some colleagues work with this type of arrangement, and then, the former one work serenely with the latter one, well, I think there is still a lot of work to do on this issue”.*

(Vittoria–Social worker, F, 41)

Remote workers and managers, who reported such reactions by colleagues, did not give too much attention to them. However, such reactions were interpreted as physiological elements of the change that the management and personnel office should consider carefully. Developing a smart working culture that includes monitoring and performance evaluation as central elements of this type of work organization, cannot be overlooked [2]. The health emergency and the experience of the emergency teleworking have, anyway, contributed to create an awareness of the effectiveness of remote working; such awareness may be a fertile ground for the development of a more solid and accepted smart working culture.

## 7. Discussion

The case study here described reports the first attempt, in the pre-COVID-19 era, to introduce a remote work program in an Italian municipality. Despite the small number of employees involved in the pilot program, this case study describes the introduction of a planned remote work program in an Italian public institution, in a period in which no emergency was imposing forced or sudden changes. Among the most notable elements of the steps followed for the implementation of the program, the following two themes are common to all the steps: participation and phasing. From the beginning, there was a tendency to involve as many people as possible. Although a limited number of positions for the pilot test were available, each employee of the municipality could apply. Those with the greatest needs were selected, while, at the same time, there was attention to select only one worker from each department. The goal was to test remote work without depriving the departments of too many employees.

Participation and phasing also characterized other choices, such as when to work outside the office, which tasks to carry out in remote work, and which criteria to use to monitor the remote work. Participation was important when, during the work process, some of those criteria were gradually modified. The practice of co-identification and co-construction of work activities and monitoring is coherent with the recent conceptualizations of work, represented as more horizontal and less hierarchical than in the past [29]. These practices provide a new perspective on public administration, because they address collaboration and joint decision-making, rather than hierarchy and super-ordination.

The human resources office played an essential role in the pilot test that is described here. It acted as a change agent and coordinator of the whole project, also taking care of the different aspects of the project. Many detailed aspects of the implementation of a remote work project were probably only marginally considered when organizations had to set up the emergency remote work at the start of the pandemic. However, such aspects will be relevant again in the regular, non-emergency remote work (e.g., setting up employer-provided IT systems, work safety procedures, legal issues, and so on).

The scientific literature and experience suggest that, in some cases, department heads or managers hinder the adoption of agile work, mainly because they are reluctant to supervise employees who are elsewhere [15,30]. For this reason, despite the good results of the described experimentation, this study enforces the fact that human resources officers play an essential rose in overcoming the possible challenges when remote work will be no longer be a necessity, but an organizational choice. The HR officers have to create a positive climate of acceptance about remote work, by highlighting its positive aspects and taking care of managers’ concerns. They also have to provide methods and procedures that managers can use to monitor work progress and ensure that employees in remote work maintain the same level of quality and efficiency as when they are in the office.

As noted by Taskin and Devos [31], and as revealed by the results of this study, remote work gives, to the human resources management, the role of coordination of the processes of negotiation and co-construction between employees and managers. Less autonomous workers, or those who do not have the necessary skills to manage or negotiate smart working, need to be helped by the HR office to benefit from this possibility to the same extent as other colleagues. In other words, the HR office is called upon to create the conditions so that remote work becomes an opportunity for most employees, also considering that previous research shows that some groups, such as single or unmarried employees, might consider unfair policies mainly benefitting family needs [32]. Furthermore, employees who are eligible for remote work, but unauthorized to it by the supervisor, might show signs of discontent, such as turnover, and should be included, when possible, in these types of programs [15,33], even when the pandemic no longer requires the use of forced remote work programs.

### 7.1. Practical Implications for HR Officers and Managers in the Post-Pandemic Era

When the pandemic ends, choosing the employees to assign to the remote work program will not be an easy process for HR officers. During this process, HR officers should avoid perceptions of unfairness within the organization.

Particular groups of employees, such as, for instance, disabled people [3], may find this work arrangement a relief from many difficulties, and the same may happen for older employees, who are more susceptible to health impairments. Thus, training programs on digital tools, video conferencing software, and safety procedures when working remotely, should be arranged and provided to employees interested in remote work.

The HR officers are also called upon to help agile workers and managers to develop communication systems to ensure productive interactions [34]. Remote work can have negative impacts on work-related knowledge sharing [35] and lead to the potential isolation of employees [3,36], as also demonstrated during the pandemic [23]. In this context, HR managers have an essential role in helping to “challenge the current norms”. Furthermore, they can take initiatives to empower employees’ skills, especially those related to the technological and digital tools to work remotely [19], to communication skills when working remotely [34], and also empower managers to take a leadership style that balances task- and relationship-oriented behavior, with a weight towards relationship-oriented leadership behavior [37]. Besides, smart working can also be an exciting element for new, younger employees, attracted by organizations offering better work–life balance conditions [38].

Recent studies highlight how remote work in public administration can add value and be appreciated by civil servants [39]. However, it is unclear how many workers will decide, when the pandemic is over, whether to return to work full time at the office, and how they will assess the choices that the HR people and managers make about remote work, to guarantee organizational efficiency and effectiveness.

Recent trends suggest that remote work will not stop when the health emergency is gone [40]. The world is moving towards increasingly hybrid forms of work. The potential for remote work is concentrated among highly skilled and educated workers in some industrial sectors, occupations, and geographies. At the same time, a large part of the workforce will have limited opportunities for remote work, because of constraints of the type of tasks to be performed [40]. Thus, we expect that more and more working at the office will alternate with working at home or other locations, as did the employees described in this study. It is also crucial that HR officers work to avoid the digital divide that separates well-educated, knowledge workers from workers with lower education in lower-paid jobs [41].

In the near future, remote work will involve high percentages of workers within the same organizations. In addition, managers will probably have to think about their role, while showing attention to different groups of workers and allowing them to work remotely. If, in the described case, managers remained on-site and coordinated their workers from the office, managers will probably also be remote workers in a short time. This shift will call into question their leadership skills, which have to be adjusted to this new scenario [37], and the very existence of the middle-level manager role. The more remote work becomes a permanent phenomenon, the more employees will become accountable, and the coordination role of managers might lose, at least partially, relevance. In this sense, co-constructing tasks and providing smart workers with the tools to switch to remote working seem to go, at least, in the direction of increasing employees’ empowerment. For this reason, it can be expected that the increasing rate of remote work will also change the middle management role.

### 7.2. Limitations of the Study

For reasons of space, this study deliberately did not examine topics such as, for example, the criteria for the selection of employees who are suitable for smart working, the issue of the technological equipment and connection, the safety aspects of remote work, or the reactions of, and coordination with, colleagues of the remote workers. Furthermore, in the study, we did not consider the perspectives of employees who were not selected for the program.

This study has other limitations. First, only a limited number of remote workers were interviewed (although we interviewed the whole population of remote workers involved, at that time, in the pilot program). Second, even if it is based on factual events, the analysis of the interviews did not use a systematic procedure. Third, the case study describes the procedures that were adopted by a public administration. However, we do believe that most of the procedures might also be the same in private companies. Fourth, the study was conducted before the pandemic, when few organizations had implemented remote work. This can be considered a limitation because the situation is now quite different; thousands of employees and organizations have experienced what has been called emergency telework. However, this case study highlights the critical steps to consider when a remote work program is intentionally and carefully introduced as a planned organizational change.

## 8. Conclusions

This case study reports some of the main elements of the implementation of a remote working program in an Italian municipality. Since Italian public institutions have been less involved in this type of initiative [6], this is one of the first studies to highlight the processes involved in implementing an agile work program in this country.

This paper highlights the design of the program, the identification of work activities to be conducted remotely, and some psychological and social outcomes of a remote work experimentation, and concludes with some implications for HR managers.

While the pilot test involved a limited number of smart workers, the experimentation of smart working had a significant impact on the municipality, setting up all the components involved in the regular and broader implementation of the program. The pilot test involved many persons, as follows: top managers of the municipality, smart workers, managers and colleagues of smart workers, HR officers, lawyers, IT officers, unions, and, to a lesser extent, also citizens who, in some cases, suffered the absence of remote workers from their physical position, but, in other instances, benefited of more digital, online services.

Although the study describes the case of an organizational transformation that took place well before the COVID-19 pandemic, the accelerated adoption of agile work, because of this health emergency, constitutes an element of encouragement towards a deeper comprehension of the organizational change processes related to the implementation of remote work in the public sector. It also represents an opportunity to change some human resource management policies and reconsider the leadership style that is adopted by middle managers. The health emergency should be considered a primer to overcome the resistance to remote work and facilitate the adoption of smart working as a solution that creates value for the organization. In the transition from emergency telework to remote working, we believe that the experiences that are described in this study are resources to start to think about how to make this contingency a valuable opportunity.

## Data Availability

Not applicable.
